# The Growing Global Healthy Longevity Ecosystem

**DOI:** 10.1093/geroni/igab046.1161

**Published:** 2021-12-17

**Authors:** Thomas Seoh

**Affiliations:** Kitalys Institute, Charlottesville, Virginia, United States

## Abstract

The geroscience field has started to grow exponentially in recent decades. This in turn has led to a rapidly emerging global ecosystem of players and nodes that has radiated out into fields from clinical investigation to medical practice to capital markets and startup activity to consumer-facing goods and services, regulations, laws and policies and the general wellness-conscious public. These are still early days, and there is uncertainty and a lack of awareness about the shape and activities of this rapidly growing and evolving community. This presentation will attempt a high-level survey of the current landscape in the hope of promoting awareness and collaborations among diverse, multiple initiatives that can accelerate the field.

